# A risk prediction system for depression in middle-aged and older adults grounded in machine learning and visualization technology: a cohort study

**DOI:** 10.3389/fpubh.2025.1606316

**Published:** 2025-06-04

**Authors:** Jinsong Du, Xinru Tao, Le Zhu, Wenhao Qi, Xiaoqiang Min, Hongyan Deng, Shujie Wei, Xiaoyan Zhang, Xiao Chang

**Affiliations:** ^1^School of Health Management, Zaozhuang University, Zaozhuang, China; ^2^School of Public Administration, Hangzhou Normal University, Hangzhou, China; ^3^Department of Teaching and Research, Shandong Coal Health School, Zaozhuang, China; ^4^School of Public Health and Nursing, Hangzhou Normal University, Hangzhou, China; ^5^Department of Geriatics, Shandong Healthcare Group Xinwen Central Hospital, Taian, China; ^6^Image Center, Zaozhuang Municipal Hospital, Zaozhuang, China; ^7^Magnetic Resonance Imaging Department, Shandong Healthcare Group Zaozhuang Central Hospital, Zaozhuang, China

**Keywords:** depression, machine learning, CHARLS, risk prediction, visualization

## Abstract

**Introduction:**

Middle-aged and older adults are highly susceptible to depression. For this reason, early identification and intervention can substantially reduce its prevalence. This study innovatively proposed a visual risk prediction system for depressive symptoms and depression in middle-aged and older adults, rooted in machine learning and visualization technologies.

**Methods:**

Using cohort data from the China Health and Retirement Longitudinal Study (CHARLS), involving 8,839 middle-aged and older adult participants, the study developed predictive models based on eight machine learning algorithms, primarily including LightGBM, XGBoost, and AdaBoost. To enhance the interpretability of the XGBoost model, SHAP technology was employed to visualize the prediction results. The model was then deployed on a web platform to establish the risk prediction system.

**Results:**

Among the models, XGBoost demonstrated the best performance, achieving an average ROC-AUC of 0.69, and was ultimately selected as the predictive model for depressive symptoms and depression risk in this population. The developed risk prediction system can output the probability of users developing depressive symptoms or depression within five years and provide explanations for the prediction results, improving user accessibility and interpretability.

**Discussion:**

Rooted in China's national longitudinal cohort, this platform integrates machine learning analytics with interactive visualization, with web deployment enhancing its clinical translational value. By enabling early depression detection and evidence-based interventions for middle-aged and older adult populations, it establishes a novel health management paradigm with demonstrated potential to improve quality of life.

## Introduction

Depression emerges as one of the most prevalent mental health issues globally, bringing about profound implications for the holistic wellbeing and life quality of millions globally (1–3). As people age, they face physical health challenges, changes in living environments, and reduced social support, rendering middle-aged and older adults a high-risk group for depression (4–6). In China, the prevalence of depression among middle-aged and older adults is continuously rising, severely impacting their overall wellbeing (7). Early intervention can not only strikingly lessen the incidence of depression, but also substantially augment the quality of life as well as the happiness of middle-aged and older adults (8, 9). On that account, developing a risk prediction system for depression in middle-aged and older adults can facilitate the early identification of high-risk individuals and enable timely interventions.

As big data and artificial intelligence move ahead continually in recent years, machine learning has demonstrated enormous potential as a powerful data analysis tool in predicting disease risk (10–12). By applying machine learning algorithms, researchers can deeply explore the underlying patterns in health data and identify high-risk individuals for diseases. This early identification not only is detrimental to elevating the incidence of diseases but also offers decision support to healthcare professionals, which ultimately empowers them to develop more personalized intervention and treatment plans. For example, Alcazer et al. (13) used data from six French university hospitals to build a machine learning model that predicts leukemia subtypes grounded in routine laboratory parameters; Liu et al. (14) pioneered a model by adopting cohort data to predict the risk of kidney failure and death in patients suffering from moderate to severe chronic kidney disease, empowering clinicians with enhanced capabilities for multidimensional patient evaluation during diagnostic decision-making processes. It is evident that machine learning technology has materialized conspicuous progress in the field of disease risk prediction. For the time being, some studies have developed prediction models for depression in middle-aged and older adults by employing cross-sectional data (15, 16). Nevertheless, inherent methodological constraints of cross-sectional designs impede comprehensive insights into the temporal trajectory of disease progression. Simultaneously, current research typically divides populations into two simple categories: “no depressive symptoms” and “depressive symptoms”, failing to take into account the different levels of depressive symptoms. In practical terms, the severity of depressive symptoms is conspicuously correlated with diseases such as cognitive impairment (17). Furthermore, most existing studies stop at the stage of developing predictive models, which are usually only accessible through professional programming platforms like Python and lack sufficient user-friendliness. Providing that a user-friendly predictive web platform or client were developed on this basis, we convince it would dramatically strengthen the practical applicability of the research.

On that account, our study utilized data from the China Health and Retirement Longitudinal Study (CHARLS) database to develop a depression risk prediction system for middle-aged and older adults. We adopted a diverse spectrum of machine learning algorithms, encompassing LightGBM, XGBoost, and AdaBoost, to construct the models and selected the best-performing model as the depression risk prediction model for middle-aged and older adults. In particular, the model can predict the risk of developing depression symptoms and depression in the next 5 years. Moreover, we utilized Shapley additive explanations (SHAP) for visual interpretation of the model to heighten the model's transparency and aid healthcare professionals in understanding the predictions (18–21). Subsequently, we innovated the model on a web platform, serving as a more actionable tool for everyday use (22). Through this system, healthcare professionals can quickly identify high-risk middle-aged and older adults for depression, enabling timely personalized intervention and management. This far-sighted risk prediction and management approach offers diverse options for depression prevention and control in public health, demonstrating massive potential for further enhancement through clinical trials in the future.

## Methods

### Study population

**Figure 1:**
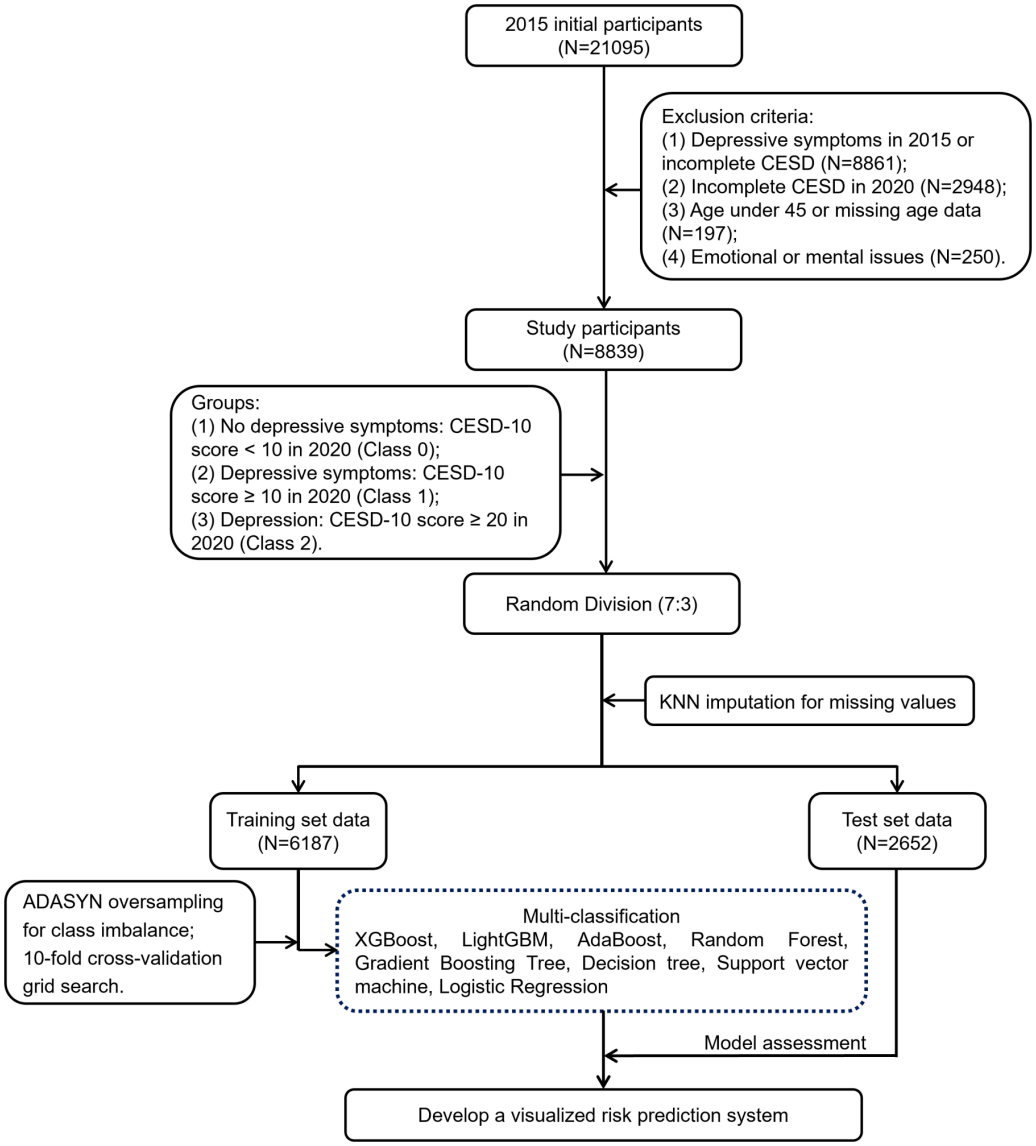
A flowchart describing the general framework of the study.

The data used in this study were sourced from the China Health and Retirement Longitudinal Study (CHARLS) (23, 24). CHARLS is a large-scale interdisciplinary survey project led by the National School of Development at Peking University, covering 28 provinces, 150 counties, and 450 communities (villages) across China. The survey collected high-quality longitudinal data through in-home visits from a nationally representative sample of individuals aged 45 and above, along with their spouses. The study was approved by the Ethics Review Committee of Peking University (IRB00001052-11015) and all participants signed informed consent forms before the study commenced. We strictly adhered to ethical principles throughout the research process, ensuring transparency and integrity and conducted all experiments in accordance with relevant guidelines and regulations. As shown in Figure 1, all samples in this study were selected from the third wave (2015) and the fifth wave (2020) of CHARLS data, ultimately including 8,839 participants. To ensure that only new cases are assessed, this study excluded participants who had already shown depressive symptoms at baseline. The exclusion criteria were as follows: (1) participants who had depressive symptoms in 2015 or did not complete the depression scale; (2) participants who did not complete the depression scale in 2020; (3) participants under the age of 45 or with missing age data; (4) participants diagnosed by a doctor with emotional or mental problems.

### Research variables

The outcome variable of depression status was assessed using the Center for Epidemiologic Studies Depression Scale (CESD-10) (Supplementary Table S1), which has shown good reliability and validity in measuring depression in middle-aged and older adults (25, 26). The scale includes 10 items, with items 5 and 8 being reverse-scored. It uses a 4-point Likert scale, with total scores ranging from 0 to 30. Based on existing research (17), in the fifth wave of the CHARLS survey, a total score of ≥10 indicates the presence of depressive symptoms, a score of ≥20 indicates depression, and a score of < 10 indicates no depressive symptoms.

This study involved a total of 69 predictor variables, categorized into five types of information: demographics, lifestyle, health status, insurance, and living environment (Supplementary Table S2), all sourced from the third wave of CHARLS data. Demographics included seven variables such as gender, age and marital status; lifestyle included 24 variables such as nightly sleep duration and marital satisfaction; health status included 24 variables such as blindness, deafness or partial deafness and muteness or stuttering; insurance included seven variables such as participation in the urban and rural residents' pension insurance; and residential environment included seven variables such as whether the housing structure was a single-story or multi-story building. Among these, the missing rate for four variables (H4, H8, L13, and L14) ranges from 5 to 8%, specifically 6.54, 5.98, 5.63, and 7.43%, respectively; while the missing rate for six variables (H1, H2, H3, L11, D3, and L17) is between 2 and 5%, specifically 3.22, 2.41, 4.53, 2.07, 4.28, and 3.41%, respectively; the missing rate for the remaining variables is below 2%.

### Prediction system development

The machine learning models in this study were constructed using Python 3.11. The dataset was randomly divided into a training set (70%) and a test set (30%) using the train_test_split algorithm, ensuring that the class proportions in the training and test sets were largely consistent with those in the original dataset. Missing values were imputed using the KNN algorithm (27), and the issue of sample distribution imbalance in the training set was addressed using Adaptive Synthetic Sampling (ADASYN) oversampling technique (28). ADASYN is an adaptive synthetic oversampling method that enhances classifier performance by generating synthetic samples similar to those in the minority class. During the oversampling process, ADASYN generated a corresponding number of synthetic samples for each minority class. The sample size for Class 1 was increased to three times the original and for Class 2, it was increased to 30 times the original. After oversampling, the final distribution of the three classes was 1:0.92:0.99, which helped alleviate the class imbalance issue. The optimal hyperparameter set was selected using 10-fold cross-validation grid search and models were constructed using XGBoost (XGB) (29), LightGBM (LGBM) (30), AdaBoost (ADA) (31), Random Forest (RF) (32), Gradient Boosting Tree (GBT) (33), Decision Tree (DT) (34), Support Vector Machine (SVM) (35), and Logistic Regression (LR) (36). Among these, XGB and LGBM are ensemble learning methods based on gradient boosting algorithms and exhibit high predictive performance; ADA and GBT perform ensemble using weighted weak classifiers; RF and DT use tree structures for data partitioning; SVM classifies by maximizing the boundary between classes; and LR uses linear models for probabilistic prediction. These eight algorithms were chosen due to their common use and representativeness (37–41). Since LR and SVM are linear models, feature scaling is required prior to their use. Therefore, in these two algorithms, we also applied the StandardScaler algorithm to standardize the data. The model performance evaluation metrics included accuracy, precision, F1-score and the area under the receiver operating characteristic curve (ROC-AUC). The best-performing model was selected as the predictor of depressive symptoms and depression risk in Middle-aged and older adults, and SHAP technology was employed to calculate the feature importance index of the optimal model, while an online prediction system was constructed (42). Furthermore, to test the robustness of the data imputation methods on the results, this study applied three imputation methods—mean imputation, mode imputation, and KNN imputation—on the data. The models were then constructed using the best-performing algorithms, and the impact of different imputation methods on model performance was tested using the Hanley-McNeil method.

## Results

### Research subjects

This study initially included 21,095 participants. Subsequent to the exclusion of 12,256 participants grounded in exclusion criteria, 8,839 participants remained in the end (Figure 1). Among these 8,839 participants, 4,717 were male (53.37%) and 4,122 were female (46.63%), with an average age of 62.6 ± 9.52 years (Supplementary Table S2). The average CESD-10 scores in 2015 and 2020 were 4.15 ± 2.79 and 6.69 ± 5.3, severally (Supplementary Table S1). A CESD-10 score of ≥20 was classified as depression, while a score of ≥10 indicated depressive symptoms. After 5 years of follow-up, among the 8,839 participants without depressive symptoms, 2,028 and 217 developed depressive symptoms and depression, separately, resulting in prevalence rates of 22.94 and 2.46%.

### Classification performance

**Figure 2:**
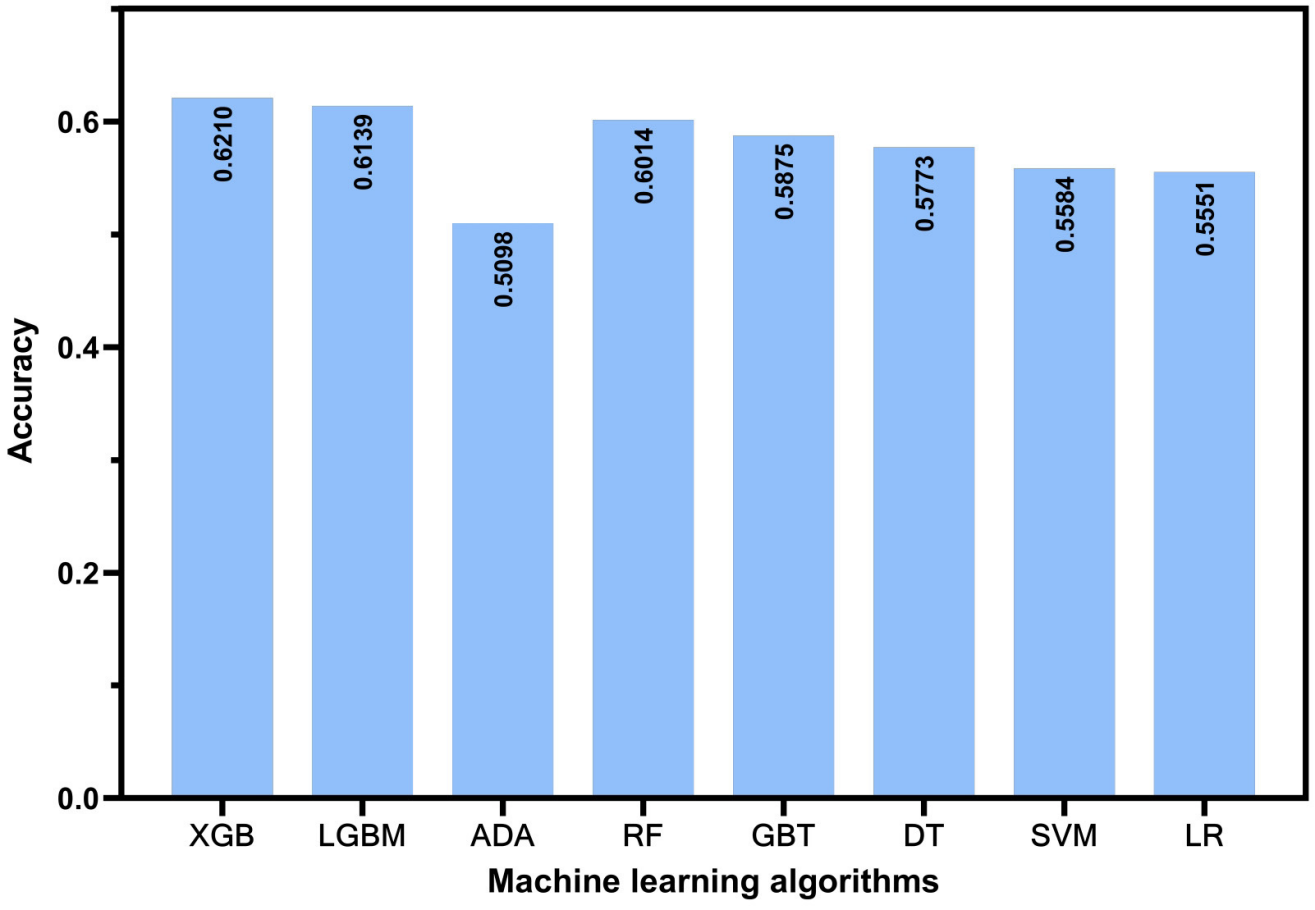
The prediction accuracy of different machine learning models.

**Figure 3:**
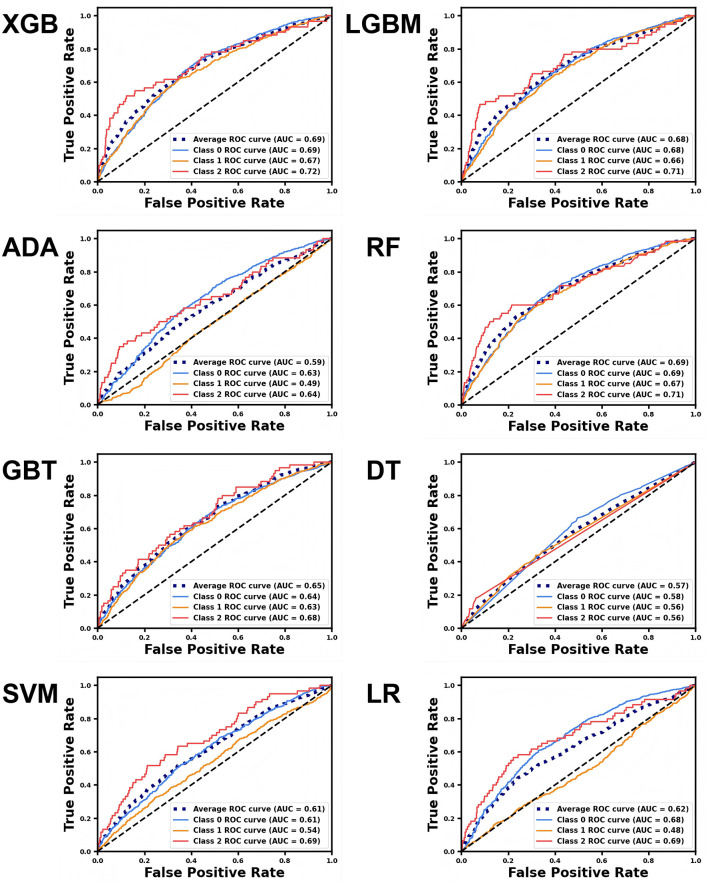
ROC curves for different machine learning models predicting various depression classes. The blue curve represents no depressive symptoms (Class 0), the yellow curve represents depressive symptoms (Class 1), and the red curve represents depression (Class 2). The average curve, represented by the blue dashed line, is the mean of the ROC curves for all three categories.

In this study, we used accuracy, precision, F1-score, and the ROC-AUC as evaluation metrics to assess models constructed by adopting eight algorithms: XGB, LGBM, ADA, RF, GBT, DT, SVM, and LR, on the basis of test set data. As illustrated in Figure 2, the models constructed by XGB, LGBM, and RF algorithms outperformed the others in terms of accuracy, achieving 0.621, 0.6139, and 0.6014, separately. Figure 3 presents the ROC curves of each model. The XGB model achieved an average ROC-AUC of 0.69, consistent with the RF model. In comparison with LGBM and RF, the XGB model also exhibited higher scores in average precision, average recall, and average F1-score (Table 1). This may be because XGB, LGBM, and RF are ensemble learning algorithms rooted in decision trees, which demonstrate more conspicuous learning and generalization capabilities when handling complex data (43). Aside from that, the XGB model iteratively optimizes the performance of each tree, effectively capturing non-linear associations and feature interactions in the data. XGB also features automatic feature selection and sample weighting, which improve its performance when handling imbalanced data and reduce the risk of over fitting (44). Considering all metrics, this study ultimately selected the model built by adopting the XGB algorithm as the predictive model for depressive symptoms and depression risk in Middle-aged and older adults. To further validate the robustness of the data imputation methods used in this study, we compared the model performance under different imputation methods. As illustrated by the aforementioned experimental findings, the ROC-AUC of the models constructed by utilizing KNN imputation was similar to those constructed by adopting mean imputation (Supplementary Figure S1A) and mode imputation (Supplementary Figure S1B), demonstrating negligible statistical difference (Supplementary Table S3).

**Table 1:** Precision, recall and F1-score of the eight machine learning models.

**Metric**	**Class**	**XGB**	**LGBM**	**ADA**	**RF**	**GBT**	**DT**	**SVM**	**LR**
Precision	Average	0.4238	0.4179	0.3536	0.417	0.3948	0.3787	0.3719	0.3936
	Class 0	0.8433	0.8332	0.7729	0.8447	0.8226	0.8038	0.7921	0.8165
	Class 1	0.3246	0.3101	0.2125	0.3174	0.2892	0.2739	0.261	0.2759
	Class 2	0.1034	0.1102	0.0755	0.0889	0.0725	0.0585	0.0625	0.0884
Recall	Average	0.5184	0.5159	0.4143	0.514	0.4255	0.4089	0.4206	0.4565
	Class 0	0.6857	0.6817	0.5697	0.6607	0.6327	0.6407	0.6282	0.5892
	Class 1	0.4196	0.3993	0.3232	0.4146	0.4772	0.4027	0.3503	0.4636
	Class 2	0.45	0.4667	0.35	0.4667	0.1667	0.1833	0.2833	0.3167
F1-score	Average	0.4302	0.4258	0.3455	0.4168	0.3921	0.3759	0.3674	0.3895
	Class 0	0.7563	0.7499	0.6559	0.7414	0.7153	0.713	0.7007	0.6845
	Class 1	0.3661	0.3491	0.2564	0.3595	0.3602	0.326	0.2991	0.346
	Class 2	0.1682	0.1783	0.1243	0.1493	0.101	0.0887	0.1024	0.1382

We probed into the classification performance of the XGB model for participants with no depressive symptoms, depressive symptoms, and depression by employing a confusion matrix (Supplementary Figure S2). Among the 2,001 participants who had no depressive symptoms 5 years later, 68.57% were correctly classified, 24.79% were incorrectly classified as having depressive symptoms, and 6.65% were misclassified as depressed. In the group of 591 participants who exhibited depressive symptoms after 5 years, 41.96% were accurately classified, while 40.95% were misclassified as having no depressive symptoms, and 17.09% were incorrectly identified as depressed. Among the 60 participants diagnosed with depression 5 years later, 45% were correctly classified, with 21.67% misclassified as having no depressive symptoms and 33.33% incorrectly classified as having depressive symptoms. The lower classification accuracy for participants with depression may be associated with the smaller proportion of this group in the dataset. Despite data balancing efforts throughout model training, the classification accuracy for this category remains lower than that of the other two categories.

### Feature significance

**Figure 4:**
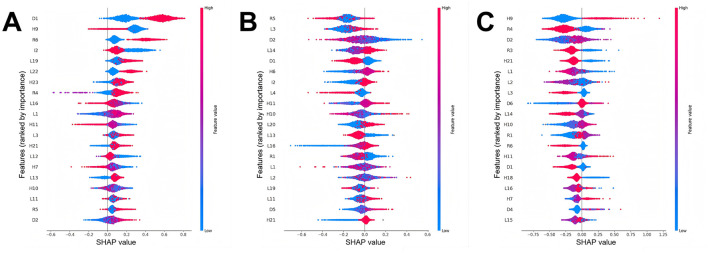
Feature importance charts for different depression classes (The detailed description of the features can be found in Supplementary Table S2). **(A)** Feature importance chart for the no depressive symptoms class, **(B)** feature importance chart for the depressive symptoms class, and **(C)** feature importance chart for the depression class.

SHAP plots are important tools for interpreting machine learning model outputs, quantifying the contribution of each feature to the model's predictions (42). In the SHAP plot, the vertical axis represents the feature significance ranking, while the horizontal axis shows the impact of each feature on the model's output. Each point in the plot defines an individual sample, with red indicating higher feature values and blue indicating lower feature values. SHAP values originate from Shapley values in game theory, which aim to fairly distribute each feature's contribution to the model's prediction. Specifically, each feature is assigned a SHAP value that denotes its contribution to predicting a particular class. A positive SHAP value suggests a positive contribution to the prediction, while a negative SHAP value indicates a negative contribution. In the overall feature significance plot for the depression category (Figure 4C), certain health status, residential environment, and lifestyle features dramatically impact the model. For example, health status features like “Are you often troubled with anybody pains?” (H9) and “How would you evaluate your health during childhood, up to and including age 15?” (H10), residential environment features like “Is there an in-house shower or bath facility? What type?” (R4) and “Does your residence have broad-band internet connection?” (R6), and lifestyle features like “During the past month, how many hours of actual sleep did you get at night (h)?” (L1), and “During the past month, how long did you take a nap after lunch (min)?” (L2) are tremendously paramount in predicting depression. As the investigation findings demonstrate, participants with poorer physical condition, worse residential environments, and less sleep are more likely to suffer from depression. In the overall feature significance plots for individuals without depressive symptoms (Figure 4A) and those with depressive symptoms (Figure 4B), features like “How would you rate your health status?” (H11) also played a crucial role. As suggested by the research findings, participants who perceive their health status as good are more likely to be free of depressive symptoms. Apart from that, participants who have access to broadband internet at home, own an electric vehicle and a computer, or frequently interact with others are also more likely to have no depressive symptoms.

### Risk prediction system

**Figure 5:**
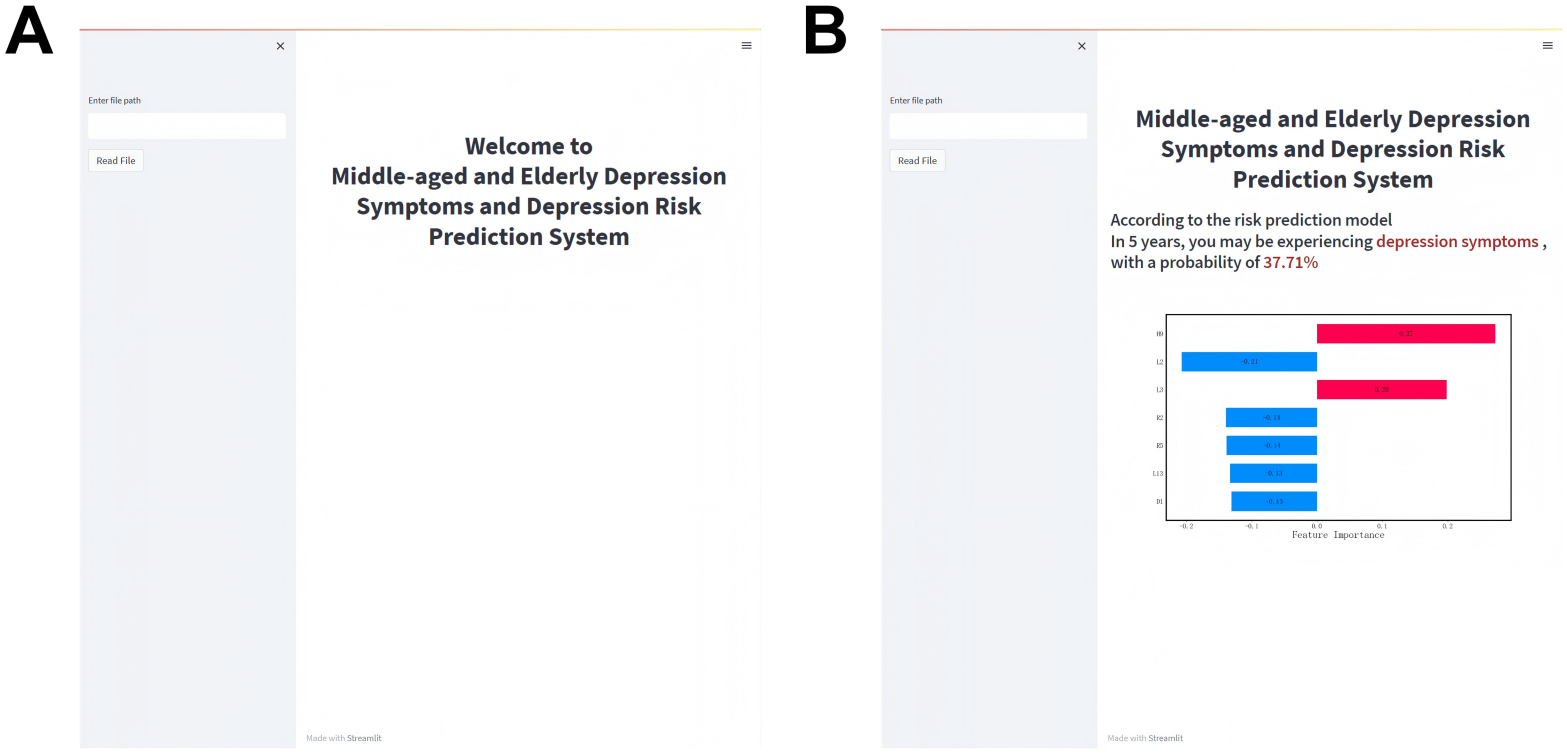
Visualization of the middle-aged and older adults depression symptoms and depression risk prediction system. **(A)** System homepage and **(B)** information output page.

In the visualization of the prediction system, the left side is the information input area where users can import data via file uploads (Figure 5). For continuous variables (e.g., age), information can be entered by dragging a slider; for categorical variables (e.g., gender), users can make selections by clicking. The right side of the system is the output window, which contains two parts. To be specific, the upper part illustrates the prediction results of the user's status after 5 years, while the lower part delivers tailored insights to inform the creation of targeted interventions. We demonstrate an example of employing the prediction system in Figure 5. After importing information in the input interface on the left, the system predicts that the user is likely to have depressive symptoms after 5 years, with a probability of 37.71%. The SHAP plot below visualizes feature contributions to predictions, with bar lengths encoding the magnitude of influence—red indicating positive contributions and blue denoting negative impacts. Features contributing positively to the predicted probability include H9 and L3, while L2, R2, R5, L13, and D1 exert negative influences. This offers fresh insights into depression prevention and treatment, suggesting that measures such as easing physical discomfort and extending sleep duration may mitigate the risk of depression among Middle-aged and older adults.

## Discussion

Grounded in longitudinal cohort data from 8,839 participants, this study successfully developed a depression symptom and risk prediction system for middle-aged and older adults, incorporating various machine learning algorithms and visualization techniques. Unlike traditional cross-sectional studies, leveraging longitudinal follow-up data spanning 2015–2020, we aimed to comprehensively map depression's developmental trajectory. We divided the population into three categories on the basis of the severity of depression: “no depressive symptoms”, “depressive symptoms”, and “depression”, overcoming the limitations of traditional binary classification models. After comparing the performance of various machine learning algorithms, the XGB algorithm was selected to build the risk prediction model, while SHAP technology was used to augment the interpretability of the model. On top of that, we developed a web-based system integrating both prediction and explanation functions, ultimately ameliorating the practicality of the research.

Above all, the primary contribution of this study lies in predicting depression symptoms and depression risk among middle-aged and older adults by adopting cohort data and machine learning algorithms. Historically, most depression prediction models in existing research have depended on cross-sectional datasets. While this approach can capture characteristics associated with depressive symptoms at a specific point in time, it cannot determine the causal association between these characteristics and depression. Cross-sectional data typically mirror the status of subjects at a single moment, which illustrates that some features may stem from changes in the outcome variable. In contrast, this study utilized longitudinal data, which effectively identified risk factors that may bring about depression, thereby offering a clearer window into depression's developmental trajectory. Apart from that, unlike previous studies (45, 46), which often categorized populations into those with and without depressive symptoms, this study further divided the population into three, namely no depressive symptoms, depressive symptoms and depression. This three-class classification approach is beneficial for identifying individuals who gradually progress from having no depressive symptoms to developing depression. Model interpretation enables granular analysis of risk factors across depressive symptom stages, yielding evidence-based guidance for tailored interventions. Furthermore, in comparison with other studies predicting depression by utilizing large cohort data, the CHARLS database included more participants (47, 48). In contrast to existing binary depression prediction studies on the basis of the CHARLS database, the prediction model exhibited a more favorable ROC-AUC value (0.61 vs. 0.69) (49).

On top of that, the study adopted SHAP technology to visually interpret the model, which is advantageous for elevating the model's interpretability. Traditional machine learning models are often seen as “black boxes” (29, 50, 51), thus rendering it challenging to understand their internal working mechanisms. Nonetheless, SHAP technology offers a way to quantify feature significance. For example, an in-depth exploration of SHAP values revealed, that a wide spectrum of factors such as pain, sleep duration, and living environment play significant roles in the model's predictions. These findings coincide with existing medical theories and clinical experience. For instance, Sanchez et al. put forward a that pain and depression share the same neurotransmitters. In addition, the presence of pain is positively correlated with the severity of depression (52). Likewise, Zambelli et al. (53) arrived at a conclusion that desirable sleep quality can strikingly ameliorate depressive states, highlighting the significance of sleep in managing depression. Furthermore, Rautio et al.'s (54) research indicated that poor housing or building environments, encompassing substandard housing quality, functional deficiencies, insufficient green spaces, as well as noise and air pollution, are remarkably associated with depressive moods. Gender (D1) also appeared in the overall feature significance charts for all three categories. As evidenced by the above findings, assuming that a participant is male, they contribute positively to the model's prediction of no depressive symptoms. On the contrary, providing that a participant is female, they positively contribute to predictions of depressive symptoms or depression. Research by Nolen et al. demonstrated that the likelihood of depression is twice as high in females as in males (55). This higher prevalence in females may be bound up with their larger share of household labor (56). Participants who frequently experience bodily pain (H9) are more prone to depression, whereas those free from such pain demonstrate a substantial tendency to be free of depressive symptoms, which corresponds with Magni et al.'s (57) perspective that pain may bring about depression. Interestingly, participants with broadband internet access at home (R6) exhibited an elevated propensity to be predicted as having no depression. As evidenced in the research conducted by Cotten et al. (58), internet use can lower depression rates among retired individuals by 20%−28%. Guo et al. (59) pointed out that improving mental health in Middle-aged and older adults is one of the welfare effects brought about by the development of internet infrastructure. Through model visualization and partial corroboration of the model's predictive logic, this research enhances clinicians' comprehension of predictive mechanisms while informing actionable clinical guidelines. More importantly, this study integrated the trained model with SHAP technology to create a depression risk prediction system for middle-aged and older adults on a web platform, eventually heightening the research's practical applicability. In the future, with ongoing refinements, anticipated implementation into standard screening protocols in primary care settings could strengthen early depression detection and intervention capabilities.

Nonetheless, this study is also less satisfactory in several aspects. To start with, despite balancing the data during model training, the accuracy of classifying the non-depressed population was ~20% higher than that of the depressed population. This phenomenon illustrates that the model's diagnostic accuracy in detecting depression remains susceptible to underlying limitations. It is essential for future research to take into account increasing the sample size of depressed patients or using cost-sensitive learning methods to elevate the model's recognition ability. Additionally, this study failed to conduct a comprehensive and profound exploration into the cost-benefit analysis of interventions rooted in model predictions. Given these considerations, a comprehensive cost-effectiveness evaluation would enhance the assessment of the prediction system's real-world applicability. Finally, this study only built the model grounded in participants' self-reported data. Although strong biological markers for depression have not yet been established, increasing data diversity may be beneficial for the reinforcement of the model's predictive ability. Future research should consider integrating different types of data to ameliorate the accuracy and reliability of the model.

## Conclusions

To sum up, this research developed a predictive framework for depressive symptoms and risk assessment in middle-aged to older adults, utilizing longitudinal cohort data from 8,839 participants and an ensemble methodology of eight machine learning algorithms. The model's interpretability was enhanced through SHAP visualization, with subsequent web-based deployment for clinical accessibility. This visualization system not only anticipates the risk of developing depressive symptoms and depression in individuals over the next 5 years but also outputs the main factors influencing the risk probabilities through SHAP plots. By enabling clinicians to interpret predictive outcomes more effectively, this system offers actionable insights for clinical practice while supporting proactive depression management. Future validation through rigorous clinical trials could further substantiate its efficacy and implementation potential.

## Data Availability

Publicly available datasets were analyzed in this study. This data can be found here: https://charls.charlsdata.com/pages/data/111/zh-cn.html.

## References

[1] Solmi M Radua J Olivola M Croce E Soardo L Salazar de Pablo G. Age at onset of mental disorders worldwide: large-scale meta-analysis of 192 epidemiological studies. Mol Psychiatry. (2022) 27:281–95. 10.1038/s41380-021-01161-7 34079068 PMC8960395

[2] Liu Q He H Yang J Feng X Zhao F Lyu J. Changes in the global burden of depression from 1990 to 2017: findings from the Global Burden of Disease study. J Psychiatr Res. (2020) 126:134–40. 10.1016/j.jpsychires.2019.08.002 31439359

[3] Yusuf S Joseph P Rangarajan S Islam S Mente A Hystad P. Modifiable risk factors, cardiovascular disease, and mortality in 155 722 individuals from 21 high-income, middle-income, and low-income countries (PURE): a prospective cohort study. Lancet. (2020) 395:795–808. 10.1016/S0140-6736(19)32008-2 31492503 PMC8006904

[4] Zhou W Chen D Hong Z Fan H Liu S Zhang L. The relationship between health-promoting lifestyles and depression in the elderly: roles of aging perceptions and social support. Qual Life Res. (2021) 30:721–8. 10.1007/s11136-020-02674-4 33068235

[5] Abdoli N Salari N Darvishi N Jafarpour S Solaymani M Mohammadi M. The global prevalence of major depressive disorder (MDD) among the elderly: a systematic review and meta-analysis. Neurosci Biobehav Rev. (2022) 132:1067–73. 10.1016/j.neubiorev.2021.10.041 34742925

[6] Ribeiro O Teixeira L Araujo L Rodriguez-Blazquez C Calderon-Larranaga A Forjaz MJ. Anxiety, depression and quality of life in older adults: trajectories of influence across age. Int J Environ Res Public Health. (2020) 17:9039. 10.3390/ijerph17239039 33291547 PMC7731150

[7] Ren X Yu S Dong W Yin P Xu X Zhou M. Burden of depression in China, 1990-2017: findings from the global burden of disease study 2017. J Affect Disord. (2020) 268:95–101. 10.1016/j.jad.2020.03.011 32158012

[8] Gilbody S Brabyn S Mitchell A Ekers D McMillan D Della B. Can we prevent depression in at-risk older adults using self-help? The UK SHARD trial of behavioral activation. Am J Geriatr Psychiatry. (2022) 30:197–207. 10.1016/j.jagp.2021.06.006 34266750

[9] Smits F Smits N Schoevers R Deeg D Beekman A Cuijpers P. An epidemiological approach to depression prevention in old age. Am J Geriatr Psychiatry. (2008) 16:444–53. 10.1097/JGP.0b013e3181662ab6 18515688

[10] Boehm KM Aherne EA Ellenson L Nikolovski I Alghamdi M Vazquez-Garcia I. Multimodal data integration using machine learning improves risk stratification of high-grade serous ovarian cancer. Nat Cancer. (2022) 3:723–33. 10.1038/s43018-022-00388-9 35764743 PMC9239907

[11] Ahsan MM Luna SA Siddique Z. Machine-learning-based disease diagnosis: a comprehensive review. Healthcare. (2022) 10:541. 10.3390/healthcare10030541 35327018 PMC8950225

[12] Du J Chang X Ye C Zeng Y Yang S Wu S. Developing a hypertension visualization risk prediction system utilizing machine learning and health check-up data. Sci Rep. 2023 13:18953. 10.1038/s41598-023-46281-y 37919314 PMC10622553

[13] Alcazer V Le Meur G Roccon M Barriere S Le Calvez B Badaoui B. Evaluation of a machine-learning model based on laboratory parameters for the prediction of acute leukaemia subtypes: a multicentre model development and validation study in France. Lancet Digit Health. (2024) 6:e323–33. 10.1016/S2589-7500(24)00044-X 38670741

[14] Liu P Sawhney S Heide-Jorgensen U Quinn RR Jensen SK McLean A. Predicting the risks of kidney failure and death in adults with moderate to severe chronic kidney disease: multinational, longitudinal, population based, cohort study. BMJ. (2024) 15:385. 10.1136/bmj-2023-078063 38621801 PMC11017135

[15] Zheng Y Zhang C Liu Y. Risk prediction models of depression in older adults with chronic diseases. J Affect Disord. (2024) 359:182–8. 10.1016/j.jad.2024.05.078 38768825

[16] Wang Y Wang X Zhao L Jones K. A case for the use of deep learning algorithms for individual and population level assessments of mental health disorders: predicting depression among China's elderly. J Affect Disord. (2025) 369:329–37. 10.1016/j.jad.2024.09.147 39321977

[17] Zhou L Ma X Wang W. Relationship between cognitive performance and depressive symptoms in Chinese older adults: the China Health and Retirement Longitudinal Study (CHARLS). J Affect Disord. (2021) 281:454–8. 10.1016/j.jad.2020.12.059 33360747

[18] Wang Y Lang J Zuo JZ Dong Y Hu Z Xu X. The radiomic-clinical model using the SHAP method for assessing the treatment response of whole-brain radiotherapy: a multicentric study. Eur Radiol. (2022) 32:8737–47. 10.1007/s00330-022-08887-0 35678859

[19] Yi F Yang H Chen D Qin Y Han H Cui J. XGBoost-SHAP-based interpretable diagnostic framework for alzheimer's disease. BMC Med Inform Decis Mak. (2023) 23:137. 10.1186/s12911-023-02238-9 37491248 PMC10369804

[20] Zheng Y Zhang Y Lu K Wang J Li L Xu D. Diagnostic value of an interpretable machine learning model based on clinical ultrasound features for follicular thyroid carcinoma. Quant Imaging Med Surg. (2024) 14:6311. 10.21037/qims-24-601 39281129 PMC11400673

[21] Nordin N Zainol Z Noor MHM Chan LF. An explainable predictive model for suicide attempt risk using an ensemble learning and Shapley Additive Explanations (SHAP) approach. Asian J Psychiatr. (2023) 79:103316. 10.1016/j.ajp.2022.103316 36395702

[22] Alabi RO Almangush A Elmusrati M Leivo I Mäkitie A. Measuring the usability and quality of explanations of a machine learning web-based tool for oral tongue cancer prognostication. Int J Environ Res Public Health. (2022) 19:8366. 10.3390/ijerph19148366 35886221 PMC9322510

[23] Zhao Y Hu Y Smith JP Strauss J. Yang G. Cohort profile: the China health and retirement longitudinal study (CHARLS). Int J Epidemiol. (2014) 43:61–8. 10.1093/ije/dys203 23243115 PMC3937970

[24] Chen X Wang Y Strauss J Zhao Y. China health and retirement longitudinal study (CHARLS). Beijing Natl School Dev. (2022) 2:948–56. 10.1007/978-3-030-22009-9_333

[25] Chin WY Choi EP Chan KT Wong CK. The psychometric properties of the center for epidemiologic studies depression scale in Chinese primary care patients: factor structure, construct validity, reliability, sensitivity and responsiveness. PLoS ONE. (2015) 10:e0135131. 10.1371/journal.pone.0135131 26252739 PMC4529142

[26] Cheng ST Chan AC Fung HH. Factorial structure of a short version of the Center for Epidemiologic Studies Depression Scale. Int J Geriatr Psychiatry. (2006) 21:333–6. 10.1002/gps.1467 16570325

[27] Beretta L Santaniello A. Nearest neighbor imputation algorithms: a critical evaluation. BMC Med Inform Decis Mak. (2016) 16:74. 10.1186/s12911-016-0318-z 27454392 PMC4959387

[28] He H Bai Y Garcia EA Li S. ADASYN: adaptive synthetic sampling approach for imbalanced learning. In: 2008 IEEE International Joint Conference on Neural Networks. IEEE (2008). p. 1322–8. 10.1109/IJCNN.2008.4633969

[29] Chen T Guestrin C. Xgboost: a scalable tree boosting system. In: Proceedings of the 22nd ACM SIGKDD International Conference on Knowledge Discovery and Data Mining. (2016). p. 785–94. 10.1145/2939672.2939785

[30] Ke G Meng Q Finley T Wang T Chen W Ma W. Lightgbm: a highly efficient gradient boosting decision tree. Adv Neural Inf Processing Syst. (2017) 30.

[31] Zhu J Zou H Rosset S Hastie T. Multi-class adaboost. Stat Interface. (2009) 2:349–60. 10.4310/SII.2009.v2.n3.a8

[32] Breiman L. Random forests. Mach Learn. (2001) 45:5–32. 10.1023/A:1010933404324

[33] Friedman JH. Greedy function approximation: a gradient boosting machine. Ann Stat. (2001) 1189–232. 10.1214/aos/1013203451

[34] Safavian SR Landgrebe D. A survey of decision tree classifier methodology. IEEE Trans Syst Man Cybern. (1991) 21:660–74. 10.1109/21.97458

[35] Noble WS. What is a support vector machine? Nat Biotechnol. (2006) 24:1565–7. 10.1038/nbt1206-1565 17160063

[36] Hosmer DW Jr Lemeshow S Sturdivant RX. Applied Logistic Regression. Vol. 398. Hoboken, NJ: John Wiley & Sons; (2013). 10.1002/9781118548387

[37] Du J Yang S Zeng Y Ye C Chang X Wu S. Visualization obesity risk prediction system based on machine learning. Sci Rep. (2024) 14:22424. 10.1038/s41598-024-73826-6 39342032 PMC11439005

[38] Du J Tao X Zhu L Wang H Qi W Min X. Development of a visualized risk prediction system for sarcopenia in older adults using machine learning: a cohort study based on CHARLS. Front Public Health. (2025) 13:1544894. 10.3389/fpubh.2025.1544894 40144970 PMC11936879

[39] King Z Farrington J Utley M Kung E Elkhodair S Harris S. Machine learning for real-time aggregated prediction of hospital admission for emergency patients. NPJ Digit Med. (2022) 5:104. 10.1038/s41746-022-00649-y 35882903 PMC9321296

[40] Abdel-Hafez A Scott IA Falconer N Canaris S Bonilla O Marxen S. Predicting therapeutic response to unfractionated heparin therapy: machine learning approach. Interact J Med Res. (2022) 11:e34533. 10.2196/34533 35993617 PMC9531006

[41] Teji JS Jain S Gupta SK Suri JS. NeoAI 1.0: Machine learning-based paradigm for prediction of neonatal and infant risk of death. Comput Biol Med. (2022) 147:105639. 10.1016/j.compbiomed.2022.105639 35635905

[42] Lundberg SM Lee S-I. A unified approach to interpreting model predictions. Adv Neural Inf Process Syst. (2017) 30.

[43] Dong X Yu Z Cao W Shi Y Ma Q. A survey on ensemble learning. Front Comput Sci. (2020) 14:241–58. 10.1007/s11704-019-8208-z

[44] Bentejac C Csorgo A Martinez-Munoz G. A comparative analysis of gradient boosting algorithms. Artif Intell Rev. (2021) 54:1937–67. 10.1007/s10462-020-09896-5

[45] Xie Y Ma M Wu W Zhang Y Zhang Y Tan X. Factors associated with depressive symptoms among the elderly in China: structural equation model. Int Psychogeriatr. (2021) 33:157–67. 10.1017/S1041610220001398 32746946

[46] Lin S Wu Y He L Fang Y. Prediction of depressive symptoms onset and long-term trajectories in home-based older adults using machine learning techniques. Aging Ment Health. (2023) 27:8–17. 10.1080/13607863.2022.2031868 35118924

[47] Andersson S Bathula DR Iliadis SI Walter M Skalkidou A. Predicting women with depressive symptoms postpartum with machine learning methods. Sci Rep. (2021) 11:7877. 10.1038/s41598-021-86368-y 33846362 PMC8041863

[48] Sampson L Jiang T Gradus JL Cabral HJ Rosellini AJ Calabrese JR. Machine learning approach to predicting new-onset depression in a military population. Psychiatr Res Clin Pract. (2021) 3:115–22. 10.1176/appi.prcp.20200031 34734165 PMC8562467

[49] Zheng Y Zhang T Yang S Wang F Zhang L Liu Y. Using machine learning to predict the probability of incident 2-year depression in older adults with chronic diseases: a retrospective cohort study. BMC Psychiatry. (2024) 24:870. 10.1186/s12888-024-06299-6 39623372 PMC11610371

[50] Linardatos P Papastefanopoulos V Kotsiantis S. Explainable AI: a review of machine learning interpretability methods. Entropy. (2021) 23:18. 10.3390/e23010018 33375658 PMC7824368

[51] Adadi A Berrada M. Peeking inside the black-box: a survey on Explainable Artificial Intelligence (XAI). IEEE Access. (2018) 6:52138–60. 10.1109/ACCESS.2018.2870052

[52] Sanchez-Rodriguez E Aragones E Jensen MP Tome-Pires C Rambla C Lopez-Cortacans G. The role of pain-related cognitions in the relationship between pain severity, depression, and pain interference in a sample of primary care patients with both chronic pain and depression. Pain Med. (2020) 21:2200–11. 10.1093/pm/pnz363 32100028

[53] Zambelli Z Halstead EJ Fidalgo AR Dimitriou D. Good sleep quality improves the relationship between pain and depression among individuals with chronic pain. Front Psychol. (2021) 12:668930. 10.3389/fpsyg.2021.668930 34025533 PMC8138032

[54] Rautio N Filatova S Lehtiniemi H Miettunen J. Living environment and its relationship to depressive mood: a systematic review. Int J Soc Psychiatry. (2018) 64:92–103. 10.1177/0020764017744582 29212385

[55] Nolen Hoeksema S. Gender differences in depression. Curr Dir Psychol Sci. (2001) 10:173–6. 10.1111/1467-8721.00142

[56] Shafer EF. Invited commentary: the uneven gender revolution and the gender gap in depression in the United States. Am J Epidemiol. (2021) 190:1207–9. 10.1093/aje/kwab003 33423056

[57] Magni G Moreschi C Rigattiluchini S Merskey H. Prospective-study on the relationship between depressive symptoms and chronic musculoskeletal pain. Pain. (1994) 56:289–97. 10.1016/0304-3959(94)90167-8 8022622

[58] Cotten SR Ford G Ford S Hale TM. Internet use and depression among older adults. Comput Human Behav. (2012) 28:496–9. 10.1016/j.chb.2011.10.021

[59] Guo HW Feng SY Liu ZM. The temperature of internet: internet use and depression of the elderly in China. Front Public Health. (2022) 10:1076007. 10.3389/fpubh.2022.1076007 36620285 PMC9811204

