# Supplementary material for: A risk prediction system for depression in middle-aged and older adults grounded in machine learning and visualization technology: a cohort study
Source: Front Public Health. 2025 Jun 4;13:1606316. doi: 10.3389/fpubh.2025.1606316 (PMC12173875; doi:10.3389/fpubh.2025.1606316)

Supplementary Material

A Risk Prediction System for Depression in Middle-Aged and Elderly Based on Machine Learning and Visualization Technology: A Cohort Study

**Supplementary Tables**

Table S1. Distribution of CESD-10 Scores.

| **CESD-10** | **2015 (mean, SD)** | **2020 (mean, SD)** |
| --- | --- | --- |
| 1. I was bothered by things that don’t usually bother me. | 0.42 (0.77) | 0.73 (0.99) |
| 2. I had trouble keeping my mind on what I was doing. | 0.41 (0.79) | 0.79 (1.03) |
| 3. I felt depressed. | 0.34 (0.66) | 0.67 (0.95) |
| 4. I felt everything I did was an effort. | 0.33 (0.72) | 0.7 (1.02) |
| 5. I felt hopeful about the future. | 1.09 (1.27) | 1.22 (1.26) |
| 6. I felt fearful. | 0.07 (0.34) | 0.26 (0.68) |
| 1. My sleep was restless. | 0.61 (1) | 0.86 (1.11) |
| 8. I was happy. | 0.69 (1.05) | 0.84 (1.11) |
| 9. I felt lonely. | 0.14 (0.5) | 0.37 (0.81) |
| 10. I could not get ”going”. | 0.05 (0.31) | 0.26 (0.7) |
| **Sum** | 4.15 (2.79) | 6.69 (5.3) |

Table S2. Baseline Characteristics of the Study Population.

| **Variables** | **Distribution** |
| --- | --- |
| **Depressive symptoms** | |
| **Class 0.** No depressive symptoms | 6594 (74.6%) |
| **Class 1.** Depressive symptoms | 2028 (22.94%) |
| **Class 2.** Depression | 217 (2.46%) |
| **Demographic** | |
| **D1.** Gender |  |
| Male (1) | 4717 (53.37%) |
| Female (0) | 4122 (46.63%) |
| **D2.** Age | 62.6 (9.52)^*^ |
| **D3.** Have you completed retirement procedures (including early retirement) or internal retirement? |  |
| Yes (1) | 912 (10.78%) |
| No (0) | 7549 (89.22%) |
| **D4.** What is your marital status? |  |
| Married with spouse present (1) | 7696 (87.07%) |
| Married but not living with spouse temporarily for reasons such as work (2) | 493 (5.58%) |
| Separated (3) | 12 (0.14%) |
| Divorced (4) | 55 (0.62%) |
| Widowed (5) | 546 (6.18%) |
| Never married (6) | 33 (0.37%) |
| Cohabitated (7) | 4 (0.05%) |
| **D5.** How many children do you have? | 2.28 (1.28)^*^ |
| **D6.** Was it village or city/town? |  |
| Main city zone (1) | 1425 (16.15%) |
| Combination zone between urban and rural areas (2) | 515 (5.84%) |
| The town center (3) | 543 (6.15%) |
| ZhenXiang area (4) | 261 (2.96%) |
| Special area (5) | 53 (0.6%) |
| Township central (6) | 114 (1.29%) |
| Village (7) | 5912 (67.01%) |
| **D7.** What is the type of the address? |  |
| Family housing (1) | 8644 (97.79%) |
| Nursing home (2) | 9 (0.1%) |
| Hospital (3) | 2 (0.02%) |
| Other (4) | 184 (2.08%) |
| **Health status** | |
| **H1.** Do you have a physical disability? |  |
| Yes (1) | 171 (2%) |
| No (0) | 8383 (98%) |
| **H2.** Do you have brain damage? |  |
| Yes (1) | 96 (1.11%) |
| No (0) | 8530 (98.89%) |
| **H3.** Are you blind? |  |
| Yes (1) | 210 (2.49%) |
| No (0) | 8229 (97.51%) |
| **H4.** Are you deaf or hard of hearing? |  |
| Yes (1) | 343 (4.15%) |
| No (0) | 7918 (95.85%) |
| **H5.** Are you mute or do you have a speech impediment? |  |
| Yes (1) | 12 (0.14%) |
| No (0) | 8812 (99.86%) |
| **H6.** How good is your eyesight for seeing things at a distance? |  |
| Excellent (1) | 325 (3.68%) |
| Very good (2) | 1602 (18.14%) |
| Good (3) | 1463 (16.57%) |
| Fair (4) | 4409 (49.93%) |
| Poor (5) | 1031 (11.68%) |
| **H7.** How good is your eyesight for seeing things up close? |  |
| Excellent (1) | 227 (2.57%) |
| Very good (2) | 1428 (16.18%) |
| Good (3) | 1582 (17.92%) |
| Fair (4) | 4308 (48.81%) |
| Poor (5) | 1281 (14.51%) |
| **H8.** Have you lost all of your teeth? |  |
| Yes (1) | 262 (3.15%) |
| No (0) | 8048 (96.85%) |
| **H9.** Are you often troubled with any body pains? |  |
| Yes (1) | 1422 (16.09%) |
| No (0) | 7414 (83.91%) |
| **H10.** How would you evaluate your health during childhood, up to and including age 15? |  |
| Excellent (1) | 1069 (12.17%) |
| Very Good (2) | 3844 (43.76%) |
| Good (3) | 1600 (18.21%) |
| Fair (4) | 1843 (20.98%) |
| Poor (5) | 429 (4.88%) |
| **H11.** How would you rate your health status? |  |
| Very good (1) | 1500 (16.97%) |
| Good (2) | 1445 (16.35%) |
| Fair (3) | 4916 (55.62%) |
| Poor (4) | 830 (9.39%) |
| Very poor (5) | 148 (1.67%) |
| **H12.** Has a doctor ever told you that you have high blood pressure? |  |
| Yes (1) | 2217 (25.13%) |
| No (0) | 6604 (74.87%) |
| **H13.** Has a doctor ever told you that you have abnormal blood lipid levels? |  |
| Yes (1) | 1085 (12.38%) |
| No (0) | 7679 (87.62%) |
| **H14.** Has a doctor ever told you that you have diabetes? |  |
| Yes (1) | 600 (6.81%) |
| No (0) | 8217 (93.19%) |
| **H15.** Has a doctor ever told you that you have cancer? |  |
| Yes (1) | 78 (0.88%) |
| No (0) | 8749 (99.12%) |
| **H16.** Has a doctor ever told you that you have chronic pulmonary disease? |  |
| Yes (1) | 669 (7.58%) |
| No (0) | 8160 (92.42%) |
| **H17.** Has a doctor ever told you that you have liver disease? |  |
| Yes (1) | 298 (3.38%) |
| No (0) | 8528 (96.62%) |
| **H18.** Has a doctor ever told you that you have heart disease? |  |
| Yes (1) | 960 (10.88%) |
| No (0) | 7862 (89.12%) |
| **H19.** Has a doctor ever told you that you have had a stroke? |  |
| Yes (1) | 104 (1.18%) |
| No (0) | 8730 (98.82%) |
| **H20.** Has a doctor ever told you that you have kidney disease? |  |
| Yes (1) | 423 (4.79%) |
| No (0) | 8405 (95.21%) |
| **H21.** Has a doctor ever told you that you have a stomach disease? |  |
| Yes (1) | 1714 (19.41%) |
| No (0) | 7115 (80.59%) |
| **H22.** Has a doctor ever told you that you have a memory-related condition? |  |
| Yes (1) | 60 (0.68%) |
| No (0) | 8767 (99.32%) |
| **H23.** Has a doctor ever told you that you have arthritis? |  |
| Yes (1) | 2346 (26.57%) |
| No (0) | 6484 (73.43%) |
| **H24.** Has a doctor ever told you that you have asthma? |  |
| Yes (1) | 236 (2.67%) |
| No (0) | 8595 (97.33%) |
| **Lifestyle** | |
| **L1.** During the past month, how many hours of actual sleep did you get at night (h)? | 6.7 (1.68)^*^ |
| **L2.** During the past month, how long did you take a nap after lunch (min)? | 39.84 (44.16)^*^ |
| **L3.** Have you visited others or socialized with friends in the past month? |  |
| Yes (1) | 3552 (40.91%) |
| No (0) | 5131 (59.09%) |
| **L4.** Have you played mahjong, chess, cards, or visited the community activity room in the past month? |  |
| Yes (1) | 2137 (24.61%) |
| No (0) | 6546 (75.39%) |
| **L5.** Have you danced, exercised, practiced qigong, or engaged in similar activities in the past month? |  |
| Yes (1) | 872 (10.04%) |
| No (0) | 7811 (89.96%) |
| **L6.** Have you participated in any club or organizational activities in the past month? |  |
| Yes (1) | 297 (3.42%) |
| No (0) | 8386 (96.58%) |
| **L7.** Have you participated in any volunteer or charity activities in the past month? |  |
| Yes (1) | 201 (2.31%) |
| No (0) | 8482 (97.69%) |
| **L8.** Have you taken care of any patients or individuals with disabilities who do not live with you in the past month? |  |
| Yes (1) | 341 (3.93%) |
| No (0) | 8342 (96.07%) |
| **L9.** Have you attended school or participated in any training courses in the past month? |  |
| Yes (1) | 128 (1.47%) |
| No (0) | 8555 (98.53%) |
| **L10.** Have you traded stocks (or funds and other financial securities) in the past month? |  |
| Yes (1) | 116 (1.34%) |
| No (0) | 8567 (98.66%) |
| **L11.** Have you ever chewed tobacco, smoked a pipe, smoked self-rolled cigarettes, or smoked cigarettes/cigars? |  |
| Yes (1) | 3894 (44.99%) |
| No (0) | 4762 (55.01%) |
| **L12.** Did you drink any alcoholic beverages, such as beer, wine, or liquor in the past year? How often? |  |
| Drink more than once a month (1) | 2755 (31.19%) |
| Drink but less than once a month (2) | 847 (9.59%) |
| None of these (3) | 5232 (59.23%) |
| **L13.** Suppose that in the future, you needed help with basic daily activities like eating or dressing. Do you have relatives or friends (besides your spouse/partner) who would be willing and able to help you over a long period of time? |  |
| Yes (1) | 2265 (27.16%) |
| No (0) | 6076 (72.84%) |
| **L14.** How satisfied are you with your marriage (relationship with spouse)? |  |
| Completely satisfied (1) | 751 (9.18%) |
| Very satisfied (2) | 3730 (45.59%) |
| Somewhat satisfied (3) | 3416 (41.75%) |
| Not very satisfied (4) | 225 (2.75%) |
| Not at all satisfied (5) | 58 (0.71%) |
| No spouse now (6) | 2 (0.02%) |
| **L15.** How satisfied are you with your relationship with children? |  |
| Completely satisfied (1) | 1016 (11.61%) |
| Very satisfied (2) | 4758 (54.39%) |
| Somewhat satisfied (3) | 2814 (32.17%) |
| Not very satisfied (4) | 141 (1.61%) |
| Not at all satisfied (5) | 17 (0.19%) |
| No child now (6) | 2 (0.02%) |
| **L16.** Please think about your life-as-a-whole. How satisfied are you with it? |  |
| Completely satisfied (1) | 709 (8.03%) |
| Very satisfied (2) | 3643 (41.28%) |
| Somewhat satisfied (3) | 4183 (47.4%) |
| Not very satisfied (4) | 270 (3.06%) |
| Not at all satisfied (5) | 20 (0.23%) |
| **L17.** Who do you think you can rely on financially for old-age support? |  |
| Children (1) | 4968 (58.19%) |
| Savings (2) | 530 (6.21%) |
| Pension or retirement salary (3) | 2770 (32.44%) |
| Commercial pension insurance (4) | 61 (0.71%) |
| Other (5) | 209 (2.45%) |
| **L18.** Do you and your spouse have a car? |  |
| Yes (1) | 1125 (12.79%) |
| No (0) | 7671 (87.21%) |
| **L19.** Do you and your spouse have an electric vehicle? |  |
| Yes (1) | 3824 (43.47%) |
| No (0) | 4972 (56.53%) |
| **L20.** Do you and your spouse have a motorcycle? |  |
| Yes (1) | 3435 (39.05%) |
| No (0) | 5361 (60.95%) |
| **L21.** Do you and your spouse have a television? |  |
| Yes (1) | 8432 (95.86%) |
| No (0) | 364 (4.14%) |
| **L22.** Do you and your spouse have a computer? |  |
| Yes (1) | 3163 (35.96%) |
| No (0) | 5633 (64.04%) |
| **L23.** Do you and your spouse have valuable furniture? |  |
| Yes (1) | 546 (6.21%) |
| No (0) | 8250 (93.79%) |
| **L24.** have you participated in vocational and technical training? |  |
| Yes (1) | 403 (4.56%) |
| No (0) | 8436 (95.44%) |
| **Insurance** | |
| **I1.** Are you participating in the basic pension insurance for employees provided by government agencies, public institutions, or enterprises? |  |
| Yes, I receive it (1) | 42 (0.48%) |
| Yes, but I don’t receive it (2) | 11 (0.12%) |
| No (0) | 8753 (99.4%) |
| **I2.** Are you participating in or receiving benefits from the New Rural Pension Insurance? |  |
| Yes (1) | 4591 (52.41%) |
| No (0) | 4169 (47.59%) |
| **I3.** Are you participating in or receiving benefits from the Urban and Rural Resident Pension Insurance? |  |
| Yes (1) | 210 (2.4%) |
| No (0) | 8550 (97.6%) |
| **I4.** Are you participating in or receiving benefits from the Urban Resident Pension Insurance? |  |
| Yes (1) | 212 (2.42%) |
| No (0) | 8548 (97.58%) |
| **I5.** Did you receive old age pension allowance? |  |
| Yes (1) | 287 (3.25%) |
| No (0) | 8534 (96.75%) |
| **I6.** Did you participate in life insurance? |  |
| Yes (1) | 703 (7.97%) |
| No (0) | 8118 (92.03%) |
| **I7.** Did you participate in commercial pension insurance, did someone buy com mercial pension insurance for you, or did you get commercial pension insurance? |  |
| Yes, but I don’t receive it (1) | 71 (0.8%) |
| Yes, I receive it (2) | 10 (0.11%) |
| No (0) | 8743 (99.08%) |
| **Residential environment** | |
| **R1.** Is the building one story or multi-level building? |  |
| One-story building (1) | 4078 (46.4%) |
| Common multi-story building (2) | 2073 (23.59%) |
| Self-contained multi-story building (3) | 2638 (30.01%) |
| **R2.** What is the type of toilet? |  |
| Toilet without a seat (1) | 6711 (76.34%) |
| Toilet with a seat (2) | 2080 (23.66%) |
| **R3.** Does your residence have running water? |  |
| Yes (1) | 7063 (80.03%) |
| No (0) | 1762 (19.97%) |
| **R4.** Is there in-house shower or bath facility? What type? |  |
| Hot water provided (1) | 95 (1.08%) |
| Water heater installed by the household (2) | 5732 (64.99%) |
| No (0) | 2993 (33.93%) |
| **R5.** Does your residence have a telephone connection? |  |
| Yes (1) | 2748 (31.14%) |
| No (0) | 6077 (68.86%) |
| **R6.** Does your residence have broad-band internet connection? |  |
| Yes (1) | 3150 (35.8%) |
| No (0) | 5648 (64.2%) |
| **R7.** Does your residence have an air cleaner in your home? |  |
| Yes (1) | 150 (1.7%) |
| No (0) | 8675 (98.3%) |

Table S3. The Hanley-McNeil test for differences in ROC-AUC between KNN imputation and mean imputation, mode imputation.

|  | *Z* | *p* |
| --- | --- | --- |
| Mean imputation | 0.257 | 0.797 |
| Mode imputation | 0.030 | 0.976 |

**Supplementary Figure**

Figure S1. The ROC-AUC of models constructed after imputing missing values using mean imputation (A) and mode imputation (B).

| A | 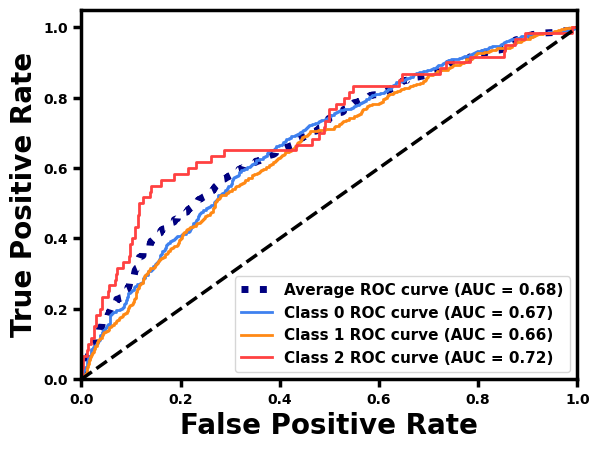 | B | 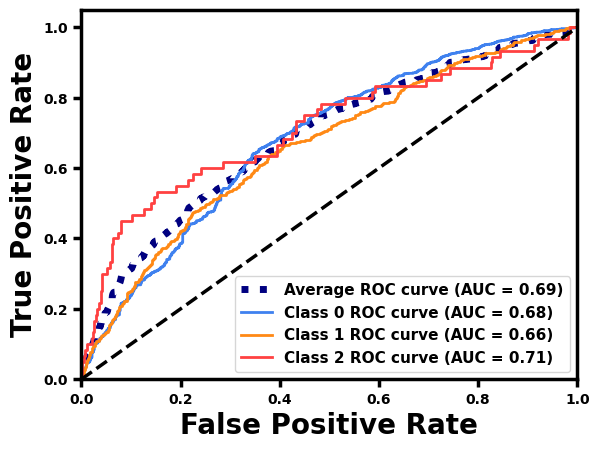 |
| --- | --- | --- | --- |

Figure S2. The mix matrix of the XGB model classification results based on the test set.


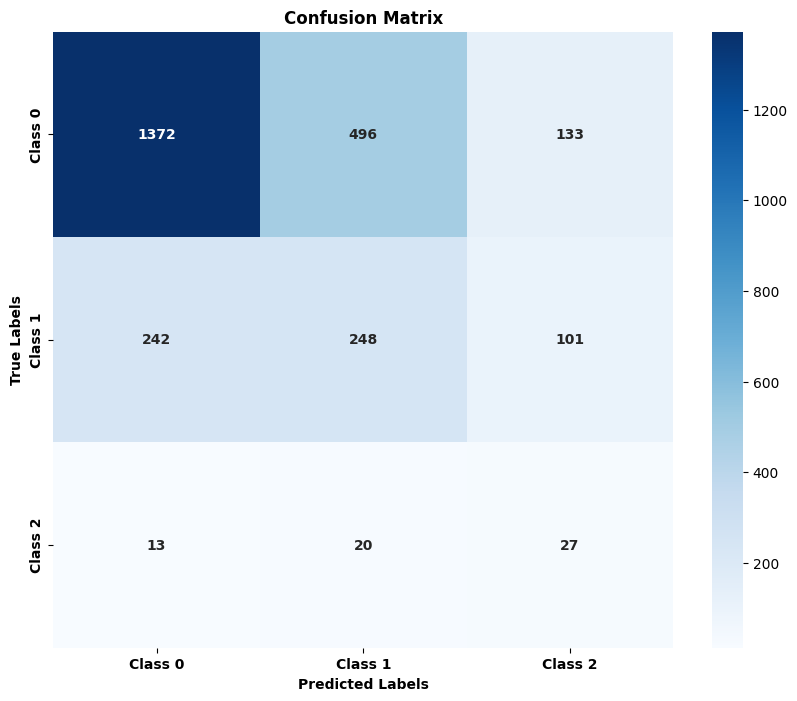

Supplement: Supplementary file 1 [file Supplementary_file_1.docx]
